# MicroRNA-31 controls G protein alpha-13 (GNA13) expression and cell invasion in breast cancer cells

**DOI:** 10.1186/s12943-015-0337-x

**Published:** 2015-03-26

**Authors:** Suhail Ahmed Kabeer Rasheed, Cui Rong Teo, Emmanuel Jean Beillard, P Mathijs Voorhoeve, Wei Zhou, Sujoy Ghosh, Patrick J Casey

**Affiliations:** Program in Cancer and Stem Cell Biology, Duke-NUS Graduate Medical School, 8 College Road, 169857 Singapore, Singapore; Program in Cardiovascular and Metabolic Disorders, Duke-NUS Graduate Medical School, 8 College Road, 169857 Singapore, Singapore; Centre for Computational Biology & Program in Cardiovascular and Metabolic Disorders, Duke-NUS Graduate Medical School, 169857 Singapore, Singapore

**Keywords:** Post-transcriptional regulation, Gα13, G12 proteins, Invasion, miR-31, Breast cancer

## Abstract

**Background:**

Gα13 (GNA13) is the α subunit of a heterotrimeric G protein that mediates signaling through specific G protein-coupled receptors (GPCRs). Our recent study showed that control of GNA13 expression by specific microRNAs (miRNAs or miRs) is important for prostate cancer cell invasion. However, little is known about the control of GNA13 expression in breast cancers. This project was carried out to determine (i) whether enhanced GNA13 expression is important for breast cancer cell invasion, and (ii) if so, the mechanism of deregulation of GNA13 expression in breast cancers.

**Methods:**

To determine the probable miRNAs regulating GNA13, online miRNA target prediction tool Targetscan and Luciferase assays with GNA13-3′-UTR were used. Effect of miRNAs on GNA13 mRNA, protein and invasion was studied using RT-PCR, western blotting and *in vitro* Boyden chamber assay respectively. Cell proliferation was done using MTT assays.

**Results:**

Overexpression of GNA13 in MCF-10a cells induced invasion, whereas knockdown of GNA13 expression in MDA-MB-231 cells inhibited invasion. Expression analysis of miRNAs predicted to bind the 3′-UTR of GNA13 revealed that miR-31 exhibited an inverse correlation to GNA13 protein expression in breast cancer cells. Ectopic expression of miR-31 in MDA-MB-231 cells significantly reduced GNA13 mRNA and protein levels, as well as GNA13-3′-UTR-reporter activity. Conversely, blocking miR-31 activity in MCF-10a cells induced GNA13 mRNA, protein and 3′-UTR reporter activity. Further, expression of miR-31 significantly inhibited MDA-MB-231 cell invasion, and this effect was partly rescued by ectopic expression of GNA13 in these cells. Examination of 48 human breast cancer tissues revealed that GNA13 mRNA levels were inversely correlated to miR-31 levels.

**Conclusions:**

These data provide strong evidence that GNA13 expression in breast cancer cells is regulated by post-transcriptional mechanisms involving miR-31. Additionally our data shows that miR-31 regulates breast cancer cell invasion partially via targeting GNA13 expression in breast cancer cells. Loss of miR-31 expression and increased GNA13 expression could be used as biomarkers of breast cancer progression.

**Electronic supplementary material:**

The online version of this article (doi:10.1186/s12943-015-0337-x) contains supplementary material, which is available to authorized users.

## Introduction

In spite of tremendous progress in cancer therapy, breast cancer remains one of the major causes of female mortality due to cancer. One of the primary reasons for this high mortality rate of this disease is the ability of breast cancer cells to metastasize to distant organs [1]. Efforts to understand the genes and signaling pathways that promote tumor invasion and metastasis have revealed that several G-protein coupled receptors (GPCRs) and their respective ligands function as metastatic drivers in breast as well other cancer types [2-6]. Some of the best-studied GPCRs are chemokine receptor 4 (CXCR4) [7], sphingosine-1-phosphate receptor 1 (S1PR1) [8], and protease-activated receptor-1 (PAR-1) [9] which are known to be upregulated in breast and other cancer types. Increased activity of CXCR4 leads to increased migration of breast cancer cells toward its ligand SDF-1 and enhanced metastasis [10]. Similarly, Thrombin-PAR1 [11] and LPA-LPAR [12] are other ligand-GPCR combinations that are highly expressed in metastatic breast cancers [3]. A common feature of all three GPCRs is that they can couple to the G12 family of heterotrimeric G proteins [13].

Heterotrimeric G-proteins, consisting of Gα, Gβ and Gγ subunits are the major effector molecules of GPCR signaling. GPCRs induce recruitment of GTP to Gα, which then are capable of activating downstream signaling pathways that lead to cell proliferation, migration, invasion and even metastasis of cancer cells [2,13-16]. All four Gα families, ie. Gq, Gi, Gs and G12, have been implicated in cancers [13,17-19]. Among these, the G12 family, consisting of Gα12 and Gα13, (the products of the GNA12 and GNA13 genes, respectively) is one of the most important in the context of cancer. Both GNA12 and GNA13 are known to be upregulated in aggressive cancer cells as well as advanced cancer tissues in several cancer types [13,20-22]. GNA12/13 proteins potentially mediate cancer cell invasion and metastasis by activating RhoA [21,23,24]. Interdicting GNA12/13 signaling using the specific inhibitor p115-RGS inhibits invasion, migration and prevents distant metastasis in mice [20,25,26]. On the other hand, expression of Gα12-QL (a dominant active mutant of GNA12) in breast and prostate cancer cells has been shown to induce *in vitro* invasion and metastatic spread in mice [20,21,27].

Most of the previous studies on the role of GNA12/13 in cancer have focused on GNA12. Recently, however, we showed that loss of wild type GNA13 alone could inhibit invasion and migration *in vitro* significantly in prostate cancer cells [28]. In the same study we reported that GNA13 was upregulated in aggressive prostate cancer cells and this upregulation was mediated by loss of microRNAs, specifically by miR-182 and miR-200a, in a synergistic fashion [28]. MicroRNAs (miRNAs, or miRs) are small non-coding RNAs that bind to the mRNA of a target gene and inhibit its protein expression. This binding of the miRNA to the 3′-UTR or coding sequence of the target gene can either lead to blocking of translation or mRNA degradation, eventually suppressing the protein production from the target gene [29]. Recently, deregulation of miRNA expression has been implicated in tumor formation and progression, wherein miRNAs can function either as ‘oncogenic-miRs’ or as ‘tumor suppressor miRs’ by targeting potential oncogenes in the cells [30]. For example, miR-21 is a well-known oncogenic-miR that targets multiple tumor suppressor genes such as PDCD4, PTEN, etc. [31]. MiR-31 is an example of a tumor suppressor miR, and is a pleotropically acting miRNA that targets multiple oncogenes such as integrin-alpha5, radixin, and EZH2 [32,33]. Most importantly, multiple studies have shown that miR-31 is lost during cancer progression and promotes metastasis of breast and other cancers [33,34].

In the current study, we found that breast cancer cells depend on GNA13 protein expression, for optimal cell invasion. Surprisingly, unlike prostate cancer cells, GNA13 expression in breast cancer cells is mainly regulated through miR-31 and not through miR-182 and miR-200a. Understanding the specific role of GNA13 in breast cancer cell invasion and the mechanism of its regulation could lead to the development of novel strategies to inhibit cancer invasion and metastasis in breast cancers using microRNAs.

## Experimental procedures

### Cell lines, reagents and plasmids

MDA-MB-231, MCF-10a, MDA-MB-157, MDA-MB-436, HMEC, and PC3 cells were purchased from Duke University Cell Repository, USA. LnCAP cells were a kind gift from Dr. Marie-Veronique Clement (National University of Singapore). HMEC cells were cultured in Clonetics™ MEGM™ Mammary Epithelial Cell Growth Medium (CC-3051). LnCAP and PC3 cells were maintained in RPMI complete media with 10% FBS and 1% Penicillin/Streptomycin (GIBCO, USA). MCF-10a cells were culture using DMEM-F12 (GIBCO, USA) supplemented with 10% FBS, 1% Penicillin/Streptomycin, 20 ng/ml EGF, 0.5 mg/ml Hydrocortisone, 10 μg/ml Insulin. The other cell lines were cultured in DMEM complete media with 10% FBS and 1% Penicillin/Streptomycin (GIBCO, USA). Matrigel inserts, plates, and growth factor-reduced Matrigel were purchased from BD Biosciences, USA. The monoclonal antibody against Gα13 (ST1629) was from Calbiochem, Germany, Gα12 antibody from Gentex (GTX114147) and antibody against α-tubulin (#010 M4813) was purchased from Sigma. Rho-A antibody was purchased from Cell Signaling (#2117). MicroRNA mimics (PremiRs) for miR-31, the PremiR-control, and antimiR-31 and the antimiR-control were purchased from Qiagen.

### Construction of GNA13-3′-UTR and miR-31-sensor-luciferase plasmids

The full length GNA13-3′-UTR (4941 base pairs) was cloned by performing a nested PCR using genomic DNA from PC3 cells using external primers (Additional file 1: Table S1). The amplified product was purified and used to amplify a 4941 base pair product using internal primers (Additional file 1: Table S1). This product was then cloned downstream to Renilla Luciferase gene (reporter) driven by 5′-LTR promoters in miR-Sens retroviral reporter vector [35] using XhoI and Not-I sites. The miR-Sens-Vector also carries a firefly luciferase gene driven by Thymidine Kinase promoter, which is used as a control to normalize the Renilla (reporter) activity in the assays as detailed previously [28] miR-31-Sensor was cloned into the same reporter as described earlier [35]. Briefly, miR-31 sensor is created by direct ligation of annealed oligos at Xho1-Not1 sites in miR-Sens vector downstream to Renilla luciferase reporter. The oligos carry a sequence complementary to mature miR-31 sequence. This will enable efficient binding of miR-31 to this sequence and suppress the Renilla luciferase activity. miR-31-Sensor’s efficiency and specificity are already tested and described in Beillard *et al.* [35]. The oligo sequences used to create miR-31 Sensor are provided in the Additional file 1: Table S1. The RhoA-3′-UTR construct was produced as described in Valastyan *et al.* and cloned in miR-Sens vector [36].

### Site directed mutagenesis of miR-Sens-GNA13-3′-UTR

The seed sequence for miR-31 at 1837–1844 within the GNA13-3′-UTR (UCUUGCAA) was mutated to UCU*GCGG*A, as shown in italic letters using specific primers carrying the mutant sequence, using QuikChange XLII™ kit according to the manufacturer’s protocol (Stratagene, USA). Briefly, the wild type miR-Sens-GNA13-3′-UTR was used as a template to amplify the mutant-31 3′-UTR using specific primers (carrying the mutations) as shown in the Additional file 1: Table S1. The parental plasmid was digested using DPN-I and the mutant plasmid was amplified by bacterial transformation. The wild type and all the mutants were verified by sequencing.

### Retroviral packaging and luciferase assays

Virus for the miR-Sens-Vector, miR-Sens -GNA13-3′UTR wt., miR-Sens-MUT-31 and miR-Sense-miR-31-Sensor was produced using the pCL-Ampho® amphotropic virus packaging plasmid in HEK293T cells as described previously [35]. Briefly, retroviruses were prepared by transfection of HEK293T cells with the 15ug of the appropriate DNA and 10ug of the packaging plasmid using lipofectamine2000™. Retroviral supernatants were harvested at 96 hours after transfection, filtered through 0.45 μM syringe filter, aliquoted and flash frozen. For basal reporter assays, cell transduction (2–5 x 10^4^ cells) was carried out with 150 μl of viral supernatant and 350 μl of media in the presence of 8 μg/ml of polybrene (Sigma) in triplicates in 24-well plates. For reporter assays with premiRs and antimiRs, 100 nM PremiRs in MDA-MB-231 cells and 100 nM antimiRs in MCF-10a cells, or control oligos, were transfected using lipofectamine2000™ 24 h post-viral transduction. Luciferase assays were performed after 48 h post-viral transduction using Dual Luciferase assay kit (Promega, USA) as described previously [35]. The Relative Light Units (RLU) were calculated using the ratio of Renilla Luciferase/Firefly luciferase and the final values were normalized against miR-Sens-vector control and the normalized values were plotted.

### Preparation of cell lysates and immunoblot analysis

Cells were seeded in a 6-well plate and transfected with premiRs, antimiRs, or control miRs (100 nM) as indicated or 1 μg pMSCV-GNA13 or shRNA-containing plasmid respectively. After 48 h of transfection, the cells were washed with phosphate-buffered saline and lysed in protein extraction-buffer (50 mM HEPES pH 7.5, 1 mM EDTA, 3 mM dithiothreitol, 10 mM MgSO_4,_ 1% polyoxyethylene-10-lauryl ether containing protease inhibitors). Protein concentration was determined by BCA protein assay (Pierce, USA). For immunoblot analysis, lysates (20 μg protein) were separated by 10% SDS–PAGE and the proteins transferred to a PVDF membrane which was incubated with the primary antibody followed by horseradish–peroxidase linked immunoglobulin G (Millipore, USA) for visualization by chemiluminescence (ECL, Thermo Scientific, USA). For stable expression of pMSCV-GNA13 or shRNAs or their respective vector controls, the cells were selected under 20 μg/ml Blasticidine for 3 weeks and lysed for western blots.

### Real-time PCR-based detection of RNA sequences

Total RNA from cells was extracted using Trizol (Qiazol, Qiagen, USA). cDNA synthesis was performed using 1 μg total RNA and the miScript-RT-II kit (Qiagen, Germany). Expression of mature miRNAs was determined using the miScript primer assays for specific microRNAs (Qiagen GmBH, Germany), and normalized using the 2^-ΔΔCT^method, using Syber Green real time PCR [37] relative to small nucleolar RNA25 (snoRD25). GNA13 mRNA was quantified using Quantitect primer assays specific for GNA13 (QT000079968) (Qiagen GmBH, Germany) using the same cDNA. Pri-miR-31 primers were taken from Yamagishi et al. [38]. PCR reactions were performed in triplicate using Quantitect Syber Green PCR mix (Qiagen, Hilden, USA). Breast cancer tissue cDNA array plates were purchased from Origene, USA (#BCRT101 – 7 Normal and 41 tumor tissue cDNA). A melting curve analysis was performed for each of the primer sets used, and each showed a single peak indicating the specificity of each of the primers tested.

### Matrigel invasion assays

MCF-10a and MDA-MB-231cells were transfected with the constructs or miRs indicated in the appropriate Figure legend using Lipofectamine. After 24 h, cells were trypsinized, and 1 x 10^5^ cells were seeded on transwell chambers precoated with 20 μg Matrigel for MDA-MB-231 cells and 10 μg Matrigel for MCF-10a cells. FCS (10%) in the lower chamber served as chemoattractant. After an additional 24 h, non-invading cells were removed with cotton swabs, and invading cells were trypsinized and counted using the Cell-Titre-Glo assay kit (Promega, USA) as described previously [39]. Remaining cells were plated into a 6-well plate for protein- and RNA-isolation after 24 h. The same method was applied to analyze MDA-MB-231 cells stably expressing shRNAs and MCF-10a cells stably expressing GNA13.

### Cell proliferation assay

250 and 500 cells respectively for MCF-10a and MDA-MB-231 were seeded in triplicates in a 96 well plate. The number of cells was determine using CellTiter 96® Aqueous one (Promega, USA) cell proliferation assay solution at 24, 48, 72, 96 and 120 hours after seeding. Briefly, 20 μl of the reagent was added to each well and incubated at 37°C for 1 hour. The absorbance reading was taken at 490 nm and plotted in the graph as shown.

### Cloning of GNA13 cDNA in pMSCV vector and construction of stable cell lines

pMSCV is a retroviral vector containing blasticidine selection marker. The cDNA encoding Gα13 cloned in pCDNA3.1 was obtained from University of Missouri cDNA Resource and subcloned into pMSCV as described in Rasheed et al. [28]. Retroviral packaging was performed as described above for pMSCV-vector control or pMSCV-GNA13 constructs. Later, 1 ml of retroviral supernatant was used to transduce 5 x 10^5^ MDA-MB-231 cells in a 10 cm dish in the presence of 8 ug/ml of polybrene (Sigma). A second transduction was performed the following day. The cells were selected using 20 ug/ml of blasticidine for 7–14 days after the second transduction. Immunoblot analysis was performed to assess the efficiency of GNA13 expression in the selected cells.

### Cloning of shRNAs and production of stable cell lines

Oligos targeting GNA13 were generated using iRNAi software for siRNA and shRNA design. The shRNAs targeting GNA13 [28] were constructed by ligating annealed oligonucleotides encoding the shRNA into a modified pRetro-Super vector at the BglII/HindIII site. The sequence of the plasmid was verified by restriction mapping and sequencing. Retroviral packaging was performed as described above for miR-sens retroviral constructs, wherein 1 ml of retroviral supernatant was used to transduce 5 X 10^5^ of MDA-MB-231 cells in a 10 cm dish in the presence of 8 ug/ml of polybrene (Sigma). A second transduction was performed the following day. The cells were selected using 20 ug/ml of blasticidine for 7–14 days after the second transduction. Immunoblots were performed to assess the efficiency of GNA13 knockdown in the selected cells.

### Statistics and online miRNA binding prediction tool

Experiments were generally performed in triplicate, and at least 3 biological replicates were performed. The significance was calculated using student’s T-test (for data pooled from all the biological replicates); a p value less than 0.05 was considered as significant. TargetScan prediction tool (http://www.targetscan.org) was used to predict the microRNA binding to the GNA13-3′-UTR, and the results validated using miRANDA, PicTar and miRwalk. Statistical analysis for real time PCR data obtained for the breast cancer tissue array samples was performed using GraphPad Prism software.

## Results

### GNA13 protein expression correlates to aggressiveness of breast cancer cells

**Figure 1:**
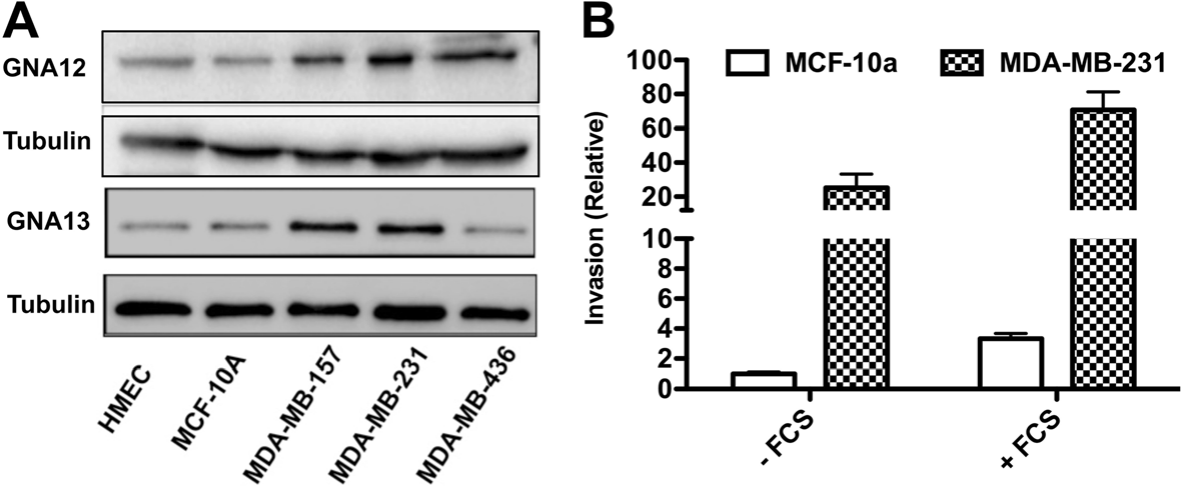
**GNA12 and GNA13 are highly expressed in aggressive cancer cells. (A)** Immunoblot showing the GNA12 and GNA13 protein expression in different breast cancer cell lines. Tubulin is used as loading control. **(B)** Basal and FCS-induced invasion of MCF-10a and MDA-MB-231 cells. All values are expressed as fold change relative to basal invasion of MCF-10a cells.

Previously others and we have shown that G12 proteins are highly expressed in prostate, breast and other cancer types [13,20,22,40]. Further, blockade of GNA12/13 signaling using the specific inhibitor p115RGS has been shown to inhibit breast and prostate cancer metastasis both *in vitro* and *in vivo* [20]. Since most of the previous studies focused on GNA12, we have recently begun to assess the specific role(s) of GNA13 in cancer cell invasion, and the control of its expression in cancers. These studies led to the above-noted findings that miR-182 and miR-200a control GNA13 expression in prostate cancer cells [28]. Similar to that observed previously in prostate cancer cell lines, examination of a panel of breast cancer cell lines revealed that GNA13 levels are greatest in more invasive cell lines such as MDA-MB-231 and MDA-MB-157 (Figure 1A). Both these cell lines were derived from metastatic breast cancers. However, more benign/less invasive breast epithelial cell lines such as MCF-10a and HMEC had much lower levels of GNA13 protein (Figure 1A). Further, the level of serum-stimulated cell invasiveness correlated with the GNA13 levels, as shown for the MCF-10a and MDA-MB-231 cell lines (Figure 1B). However, this correlation to invasiveness is only indicative of an important role of GNA13 in their invasive ability, as these cells may have multiple gene expression changes that contribute to this effect.

**Figure 2:**
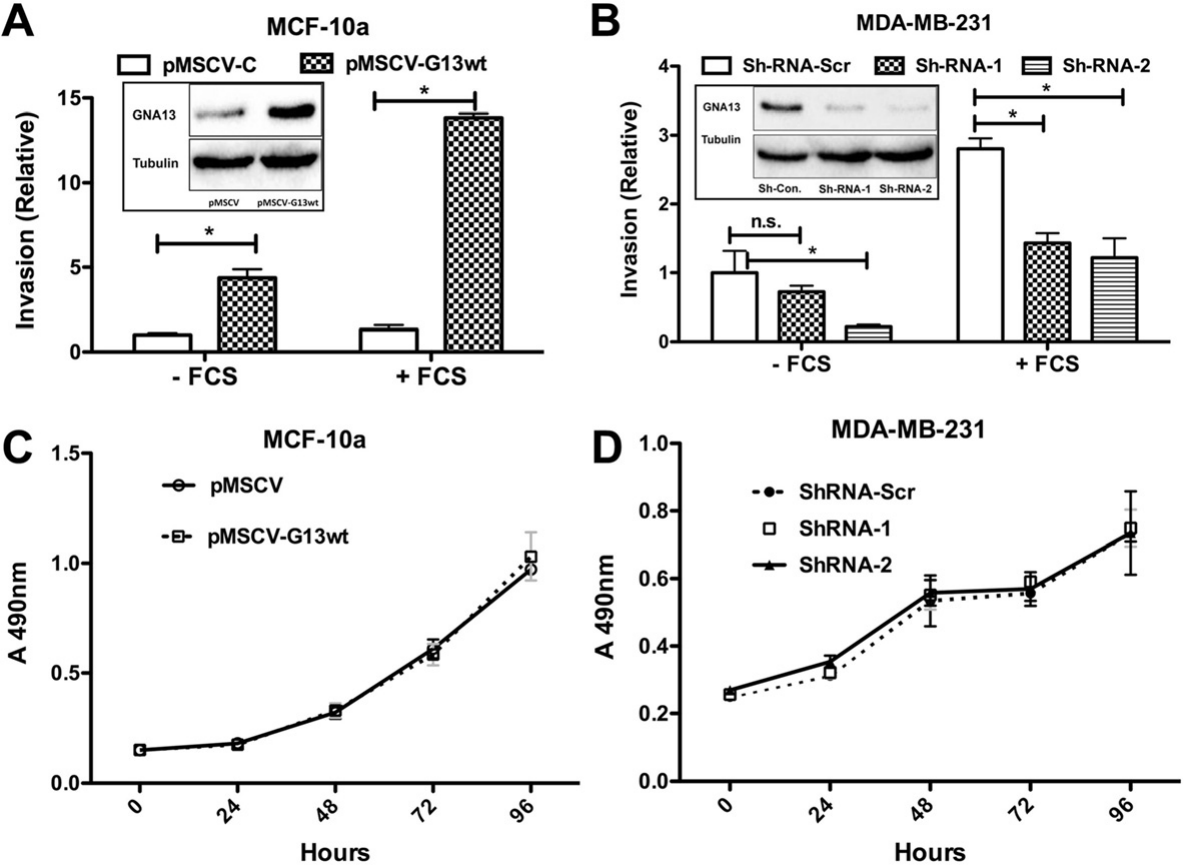
**GNA13 protein expression correlates with aggressiveness of breast cancer cells. (A)** Enforced expression of GNA13 in MCF-10a cells enhances both basal and FCS-induced invasion. Relative invasion is reported as fold change relative to pMSCV-Control (−FCS). The inset shows immunoblot analysis of GNA13 and tubulin in the same cells. **(B)** Knockdown of GNA13 protein expression inhibits basal and FCS-induced invasion of MDA-MB-231 cells. The inset shows the knockdown efficiency of GNA13. Invasion is reported as the relative fold change in relation to MDA-MB-231-Sh-Scr (−FCS). Panels **(C)** and **(D)** - cell proliferation data of MCF-10a and MDA-MB-231 cells, respectively. MTT assays were performed as described in methods and absorbance at 490 is plotted in the y-axis and hours on the x-axis as shown (*, p < 0.05).

To determine whether GNA13 played a direct role in the invasiveness and proliferation of these cells, we performed an *in vitro* invasion assay and proliferation assays. Enforced expression of GNA13 in MCF-10a cells, which normally harbor low GNA13 levels, significantly enhanced basal as well serum-stimulated invasion (Figure 2A). On the other hand, the invasion of MDA-MB-231 cells, which normally harbor high levels of GNA13 cells, was significantly inhibited when GNA13 expression was knocked down using two different shRNAs (Figure 2B). However, manipulating GNA13 expression in breast cancer cells had no effect on cell proliferation for either cell type (Figure 2C, D). Taken together, these data indicate that GNA13 is an important regulator of breast cancer cell invasion, but not cell proliferation.

### Relationship of microRNA-31 and GNA13 in breast cancer cells

Given our recent findings that GNA13 expression is regulated via post-transcriptional mechanisms in prostate cancer cells by miRNAs, we were curious as to whether similar mechanisms might be involved in control of expression of GNA13 in breast cancer cells. To explore this possibility, we first examined the predicted miRNA binding sites in the 3′-UTR of GNA13. As reported earlier [28], we identified eight miRNA binding sites in the 3′-UTR that are highly conserved across mammalian species (See schematic in Rasheed *et al.*, [28]). The miRNAs that were predicted to bind the GNA13-3′**-**UTR are miR-30 family, miR-27a/b, miR-128, miR-31, miR-182, miR-29a/b/c and miR-141/200a. It is important to note that miR-31 and miR-182 had overlapping binding sites, suggesting that either of these miRNAs could bind to the site based on the availability of the miRNA.

**Figure 3:**
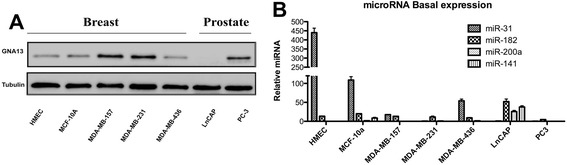
**Relationship of miR-31 and GNA13 in breast cancer cells. (A)** Immunoblot analysis of GNA13 protein expression in breast and prostate cancer cell lines. Tubulin is used as loading control. **(B)** Relative basal expression of the microRNAs predicted to bind to the GNA13-3**′**-UTR in breast and prostate cancer cells. Relative microRNA expression is plotted in the y-axis. All values were calibrated to basal miR-31 expression in MDA-MB-231 cells.

To obtain evidence for which, if any, of the potential regulatory miRNAs might be impacting GNA13 expression in breast cancer cells, we performed a comparison of GNA13 protein levels (by immunoblot analysis) and of the respective miRNAs (by real time PCR) (Figure 3A, B), respectively. Surprisingly, both miR-182 and miR-200a/141, which showed an inverse correlation to GNA13 protein expression in prostate cancer cells as reported earlier [28], were highly suppressed in breast cancer cells and hence had no correlation to GNA13 protein expression (Figure 3A, B). Interestingly, however, miR-31 showed a clear inverse correlation to GNA13 protein expression in the breast cancer cells (Figure 3A, B). Levels of this miRNA were much higher in cells containing low GNA13 protein levels such as HMECs and MCF-10a (Figure 3A, B). MDA-MB-231 and MDA-MB-157 cells expressed high GNA13 protein and had no or little detectable miR-31 expression (Figure 3A, B). These data implicate miR-31 in control of GNA13 expression in breast cancer cells.

### Mir-31 binds directly to the 3′UTR of GNA13

**Figure 4:**
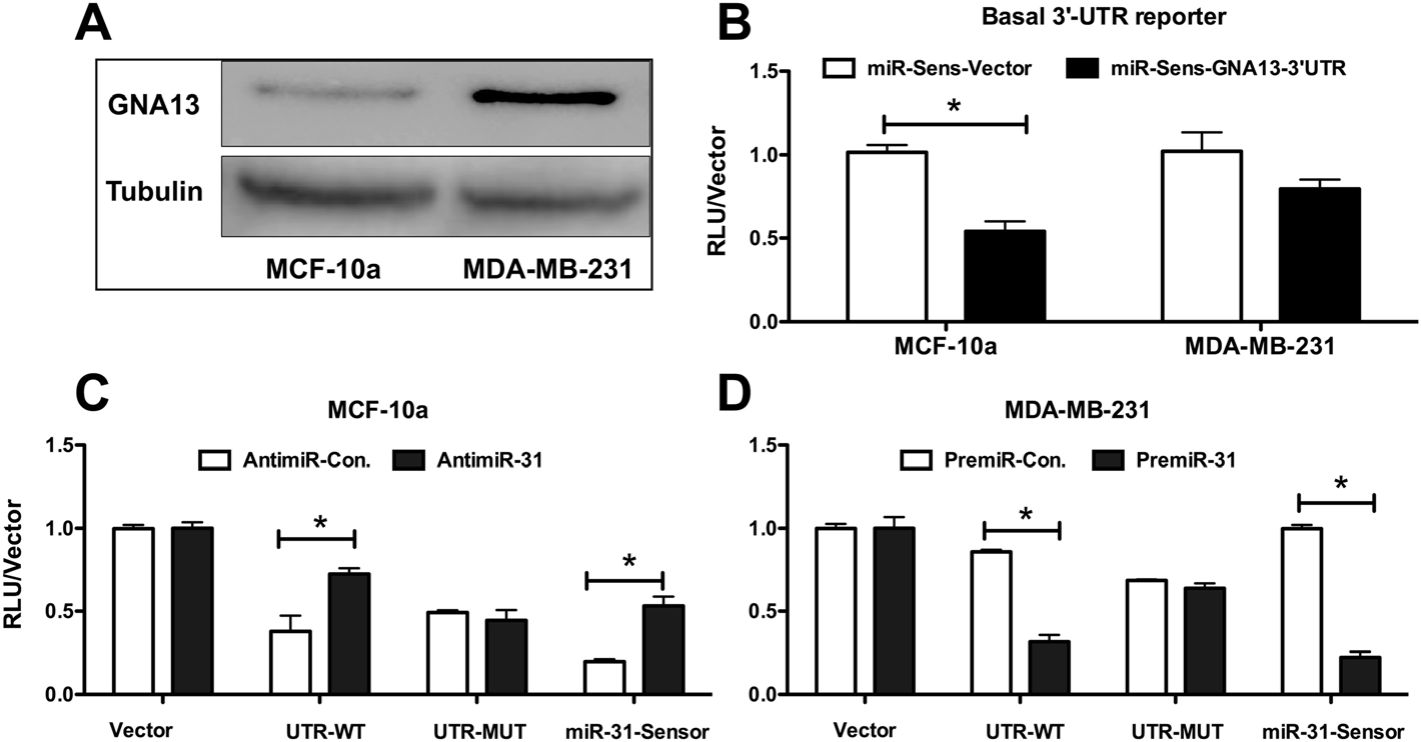
**microRNA-31directly binds to GNA13-3-UTR. (A)** Basal GNA13 protein is highly expressed in MDA-MB-231 cells relative to MCF-10a cells. Immunoblot analysis of GNA13 and tubulin expression in MCF-10a and MDA-MB-231 cells is shown. Tubulin is used as a loading control **(B)** Basal GNA13-3**′-**UTR activity correlates to GNA13 protein expression. Basal GNA13-3**′-**UTR reporter activity in MCF-10a and MDA-MB-231 cells was determined. All values are reported relative to to miR-Sens-vector reporter activity in MCF-10a cells. **(C)** Blockade of miR-31 activity using antimiR-31 rescues GNA13-3′-UTR activity in MCF-10a cells. Reporter assays were performed in MCF-10a cells transfected with antimiR-control or antimiR-31. All values are reported as fold change to miR-Sens-vector in antimiR-control treated cells. **(D)** Enforced expression of miR-31 in MDA-MB-231 cells suppresses the GNA13-3′-UTR activity in MDA-MB-231 cells. Reporter assays were performed in MDA-MB-231 cells transfected with premiR-control or premiR-31. Results are reported relative to the miR-Sens-vector control treated with premiR-control (*, p < 0.05). miR-31-Sensor is used as a positive control in the reporter assays **(C)** and **(D)**. This sensor carries a single miR-31 binding site as the 3′-UTR of Renilla Luciferase reporter. Any change in miR-31 levels is efficiently reflected as change in reporter activity. See methods for details.

To obtain evidence as to whether miR-31 binds to the 3′-UTR of GNA13 as suggested by the data presented above, we cloned the full-length UTR into miR-Sens-reporter where it controls the expression of the Renilla luciferase gene in the vector (see Figure 2B in Rasheed etal [28] for schematic of GNA13-3′-UTR). Firefly luciferase driven by thymidine kinase promoter within the same vector was used as an internal control. Transfection of these constructs into MCF-10a and MDA-MB-231 cells and measurement of reporter activity revealed that the basal activity of the GNA13-3′-UTR was inhibited significantly in MCF-10a cells, while no significant inhibitory activity was observed in MDA-MB-231 cells (Figure 4B). Further, the basal reporter activity correlated with the basal GNA13 protein expression in these cells (Figure 4A, B). This data indicate that MCF-10a cells express an inhibitory element toward the 3′-UTR, and that this element is absent or reduced in the MDA-MB-231 cells. To determine whether miR-31 was the inhibitory element expressed in MCF-10a cells, antimiR-31 was co-transfected with the GNA13-3′-UTR into the cells. Inhibition of miR-31 activity by such enforced expression of antimiR-31 largely rescued the GNA13-3′-UTR and the miR-31 sensor (a reporter carrying miR-31 binding site [35]) activity in these cells (Figure 4C). Consistent with this finding, transfection of premiR-31 into MDA-MB-231 cells suppressed the activities of both the GNA13-3′-UTR and the miR-31 sensor (Figure 4D). Further, when the predicted binding sequence for miR-31 in the GNA13-3′-UTR was mutated; neither antimiR-31 nor premiR-31 had any effect on the reporter activity (Figure 4C, D). This data provide compelling evidence that miR-31 directly binds to the GNA13-3′-UTR and inhibits its activity.

### MiR-31 activity reduces GNA13 mRNA and protein expression and inhibits breast cancer cell invasion

**Figure 5:**
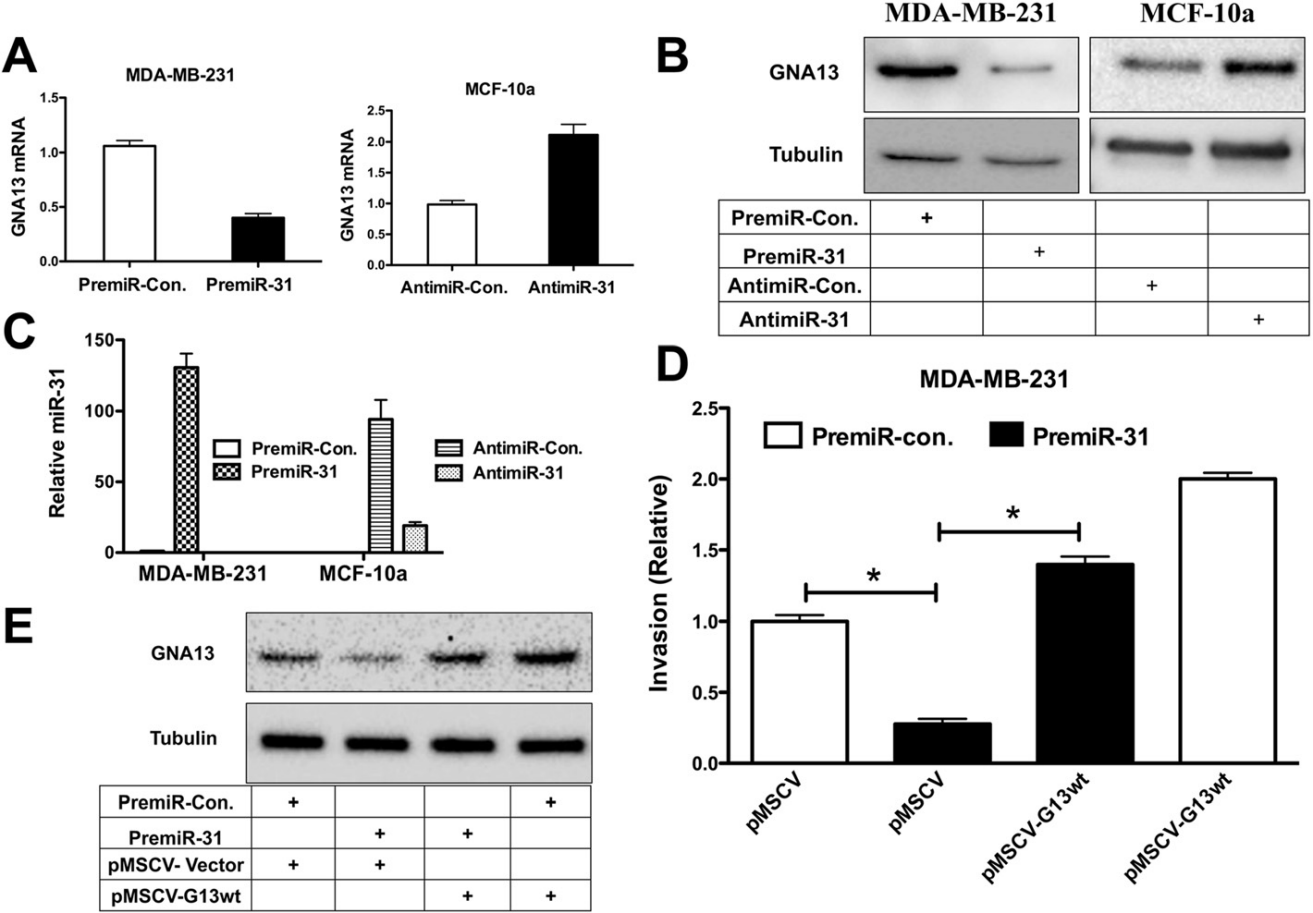
**miR-31 inhibits GNA13 expression and cancer cell invasion.** Altering miR-31 expression or activity impacts GNA13 mRNA and protein expression in MDA-MB-231 and MCF-10a cells. **(A)** GNA13 mRNA expression is reported relative to premiR-control or antimiR-control treated cells, respectively. **(B)** Immunoblot analysis of GNA13 and tubulin expression following treatment with premiRs or antimiRs as indicated. **(C)** miR-31 levels in MDA-MB-231 and MCF-10a cells following treatment with premir-31 and antimir-31, respectively. Values reported are relative to miR-31 levels in MDA-MB-231 cells treated with premiR-control. **(D)** PremiR-31 transfection inhibits invasion of MDA-MB-231 breast cancer cells. Invasion is reported as fold change relative to invasion of premiR-control + pMSCV-vector transfected cells. (*, p < 0.05). **(E)** Immunoblot analysis of GNA13 and tubulin levels in the MDA-MB-231 cells from **(D)**.

All of the data presented to date are consistent with the notion that the GNA13-3′-UTR is a target of miR-31 activity in breast cancer cells. To determine whether, miR-31 is actually involved in the regulation of expression of GNA13 in these cells, we transfected MDA-MB-231 cells with premiR-31, and MCF-10a cells with antimiR-31, respectively. MDA-MB-231 cells transfected with premiR-31 showed a significant reduction in both GNA13 mRNA and protein expression when compared with cells treated with premiR-control alone (Figure 5A, B). In addition, inhibition of miR-31 expression using antimiR-31 in MCF-10a cells resulted in a significant increase in GNA13 mRNA and protein levels (Figure 5A, B). Relative miR-31 expression after transfection with premiR-31 and antimiR-31 respectively is shown in Figure 5C. miRNA binding to the 3′-UTR of an mRNA can suppress its expression either by blocking translation or by inducing degradation of the mRNA. Hence, in addition to demonstrating that miR-31 directly binds to the GNA13-3′-UTR and inhibits GNA13 expression, these data suggest that miR-31-induced degradation of the mRNA may play a role in this process.

A previous report showed that enforced expression of miR-31 inhibits invasion and metastasis of breast cancer cells, and also that this miRNA is suppressed in metastatic breast cancers [36]. Consistent with this prior finding, transfection of premiR-31 suppressed serum-stimulated invasion of MDA-MB-231 cells (Figure 5D); GNA13 protein was also reduced by this treatment (Figure 5E). Importantly, miR-31-induced suppression of invasion was significantly rescued by enforced expression of GNA13 in premiR-31 treated cells (Figure 5D, E). These data collectively indicate that miR-31 inhibits invasion of MDA-MB-231 cells and that this is at least in part mediated through an impact on GNA13 expression.

### Relationship of miR-31 and GNA13 in breast cancer tissues

**Figure 6:**
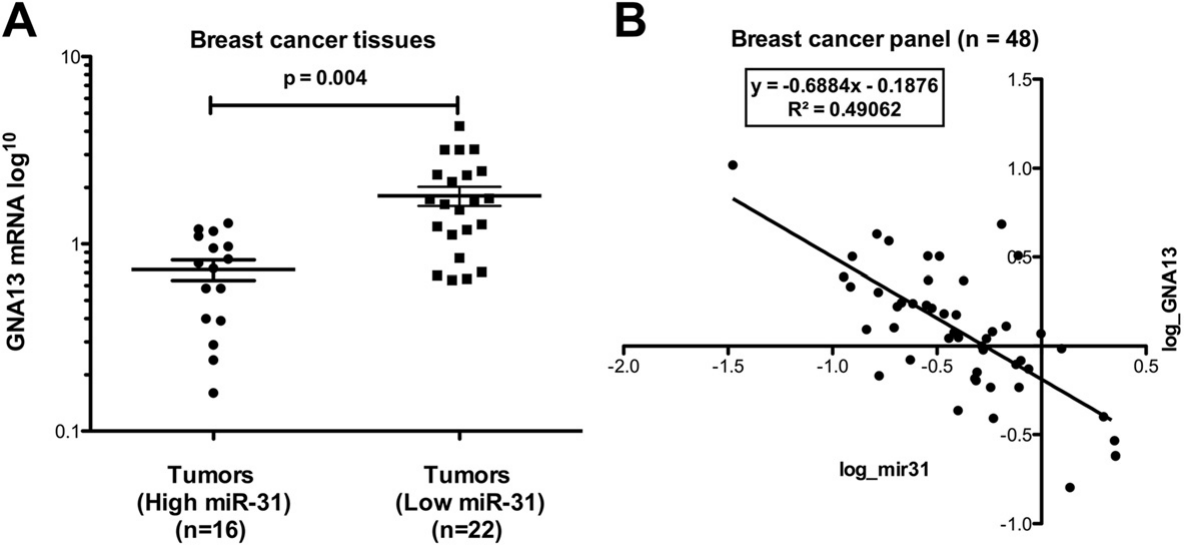
**Relationship of miR-31 and GNA13 in breast cancer tissues.** GNA13 mRNA expression shows an inverse correlation to pri-miR-31 expression in breast cancer tissues. Real time PCR was performed in a breast cancer array of 48 samples using specific primers for Pri-miR-31 and GNA13. **(A)** Tumor tissues were grouped into two groups based on their Pri-miR-31 expression relative to normal tissues. GNA13 mRNA expression in the respective tissues is plotted in the y-axis in log scale as shown. **(B)** An XY plot showing relative Pri-miR-31 expression in the x-axis and relative GNA13 expression in the y-axis, on log scale (correlation = −0.7) (*, p < 0.05).

The data presented above provide compelling evidence that miR-31 directly binds to GNA13 mRNA, and that this binding impacts both GNA13 expression and breast cancer cell invasion. These findings raise the question as to whether this process is important in the actual progression of human breast cancer. To begin to address this question, we performed an analysis of mRNA levels of Pri-miR-31 (primary transcript of miR-31) and GNA13 in 7 normal breast and 41 breast tumor tissues. The tissues were divided into two groups based on their Pri-miR-31 expression as miR-31 high (n = 16) and miR-31 low (n = 22), respectively. Comparing the basal GNA13 mRNA expression in these two groups showed that miR-31 high tissues had significantly less GNA13 mRNA as compared with miR-31 low tumors (Figure 6A). Most importantly, a correlation analysis of miR-31 expression to GNA13 expression showed a significant inverse correlation, as shown in Figure 6B. These data provide evidence that microRNA-31 might indeed be one of the determinants of GNA13 expression in breast cancers.

## Discussion

GPCRs are one of the largest classes of cell surface receptors and have pivotal roles in many physiological processes. Several GPCRs and their ligands, e.g. SDF-1, thrombin, LPA, S1P, and endothelin, have been implicated in tumor formation and organ-specific metastasis in prostate, breast, and other cancers [3]. A common feature of these GPCRs is that they can signal through G12 proteins, and indeed signaling through these proteins has been shown to impact cancer cell invasion and metastasis [13,25,41-44]. Our findings reported here that GNA13 is most highly expressed in more aggressive breast cancer cells, and that knockdown of GNA13 in these cells suppresses invasion, indicate that breast cancer cells, like prostate cancer cells [28], depend on GNA13 expression for their ability to invade in response to particular GPCR ligands. However, there are some exceptions to these observations. For example, one of the cell lines in our breast cancer screen, MDA-MB-436, had relatively low expression of GNA13 even though this cell line is derived from metastatic breast cancer. This indicates that not all cells depend on GNA13 for their invasiveness, and there are alternative pathways that might contribute to invasion and metastasis in some cell types.

Recently it has become clear that an important post-transcriptional control mechanism for gene expression is via miRNAs [30,45]. Additionally, deregulation of miRNAs has been closely associated with oncogenesis and tumor progression in several cancer types [30,45], In the context of GNA13 regulation, we have reported that miR-182 and miR-200a are involved in control of GNA13 expression in prostate cancer cells [28]. Based on these observations, we performed a similar analysis in a panel of breast cancer cells. To our surprise, both miR-182 and miR-200a are highly suppressed in all the breast cancer cells tested, irrespective of the endogenous GNA13 protein expression in the cell type. However, analysis of the basal expression of other miRNAs that are predicted to bind to GNA13-3′-UTR revealed that miR-31 showed an inverse correlation to GNA13 expression in these cells, and further studies showed that miR-31 was indeed was involved in control of GNA13 levels in the breast cancer cells. These finding reinforce the notion [46] that the same gene can be regulated by different microRNAs in different cell types based on the availability of the microRNA (46).

In addition to suppressing GNA13 expression, we observed that miR-31 suppresses invasion of MDA-MB-231 cells, and that this could be partially rescued upon ectopic expression of miR-31-resistant GNA13 in the cells. This partial rescue of miR-31-induced suppression of invasion reinforces the notion that miR-31 has multiple targets that contribute to invasion of these cells. Specifically in this regard, miR-31 has been implicated in targeting multiple genes linked to cell invasion, e.g., integrin-alpha5, radixin, RhoA, EZH2, PRKCE and LATS2 [32,33,35,47]. Since GNA13 is known to promote invasion mainly through activating RhoA [28], we also studied the impact of miR-31 on RhoA expression. However, in our system miR-31 neither targeted the RhoA-3′-UTR, nor did RhoA protein expression correlate to miR-31 expression in these breast cancer cells (Additional file 1: Figure S1 and Figure S2).

Previous reports have shown that miR-31 expression is lost during cancer progression in breast and other cancer types, either by genetic or epigenetic mechanisms [33,48]. In our screen for the expression of miR-31 and GNA13 mRNA in panel of 41 breast tumor tissues, mRNA levels for miR-31 showed an inverse correlation to those of GNA13. These data reinforce a previous finding that loss of miR-31 might be an important determinant in breast cancer metastasis [33,36], and further suggest that gain of GNA13 expression might be one of the important contributors to the phenotype. Hence, identifying the specific mechanism(s) of GNA13 regulation in breast and other cancers could potentially lead to the development of miRNA-based therapeutic strategies for these cancers. Further, loss of miR-31 and gain of GNA13 may be viable biomarkers for assessment of breast and other cancers.

## Conclusions

Our data demonstrate that GNA13 is an important mediator of cancer cell invasion, and that its expression is regulated by microRNA-31 in breast cancer cells. A screen for microRNAs that are predicted to target GNA13 in breast cancer cells revealed that miR-31 shows an inverse correlation to GNA13 protein expression in these cells. GNA13-3′-UTR activity assays showed that miR-31 directly binds to the 3′-UTR and suppressed its activity. In addition, altering the endogenous miR-31 levels using premiR-31 or antimiR-31 significantly altered GNA13 mRNA and protein expression in MDA-MB-231 and MCF-10a cells respectively. In the highly invasive cell line MDA-MB-231, which expresses high levels of GNA13, premiR-31 transfection inhibited GNA13 expression and its invasion and this could be partially rescued by ectopic expression of GNA13. Importantly a screen for miR-31 and GNA13 expression showed a significant inverse correlation of these two transcripts in breast cancer tissues. Taken together, our data indicates that loss of miR-31 in breast cancers leads to increased GNA13 expression and cancer cell invasion.

## Additional file

Additional file 1:
**Table S1.** Primers used in the cloning of GNA13-3′-UTR and for site-directed mutagenesis of miR-31 binding sites within the UTR. Oligos used to clone miR-31-Sensor in miR-Sens vector as described in Beillard *et al.* **Figure S1.** RhoA protein expression in breast cancer cells: RhoA protein expression showed no correlation to basal miR-31 expression in breast cancer cells (see Figure 3C for miR-31). Immunoblot analysis of RhoA protein levels in a panel of five different breast cell lines is shown. Tubulin was used as loading control. **Figure S2.** microRNA-31 did not impact RhoA-3′-UTR: (A) Reporter assays performed in MCF-10a cells transfected with antimiR-control or antimiR-31. (B) Reporter assays performed in MDA-MB-231 cells transfected with premiR-control or premiR-31. All reporter assays results are reported relative to miR-Sens-vector treated with antimiR-control or premiR-control respectively. miR-31 sensor is used as a measure of miR-31 activity with and without premiR-31 and antimiR-31 respectively (*, p < 0.05).
