# Supplementary material for: MicroRNA-31 controls G protein alpha-13 (GNA13) expression and cell invasion in breast cancer cells
Source: Mol Cancer. 2015 Mar 26;14:67. doi: 10.1186/s12943-015-0337-x (PMC4379695; doi:10.1186/s12943-015-0337-x)

Supplementary Data

Supplementary Table 1	Primer sequences
	**GNA13-3'-UTR-miR-Sens cloning - nested primers**
**External Fwd**	5'-TCTGCATGACAACCTCAAGC-3'
**External Rev**	5'-TTGAATTGTTTACAAATGTTTATTAAATGTC-3'
**Internal Fwd**	5'- ATCGCTCGAGTGTACAAAAGACTTGCTGTTTTAATATCTT-3'
**Internal Rev**	5'- ATCATATGCGGCCGCAAATGTCAGTAATTTTTACAAAGCAAA-3'
	**Oligos for miR-31_Sensor-miR-Sens cloning (miR-31 seed underlined)**
**miR-31_Sensor-Fw**	5'-TCGAG<u>AGCTATGCCAGCATCTTGCCT</u> GC-3'
**miR-31_Sensor-Rv**	5'-GGCCGC <u>AGGCAAGATGCTGGCATAGT</u> C-3'
	**GNA13-3'-UTR- site directed mutagenesis of miR-31 site**
**miR-31-Mut-Fwd**	5'-GCGTGTTTGAATAGTTCTACATCTAGTGCT TCTGCGGAAAAAGAATACATTGTTTAAATTCACAAAATTA-3'
**miR-31-Mut-Rev**	5'-TAATTTTGTGAATTTAAACAATGTATTC TTTTTCCGCAGAAGCACTAGATGTAGAACTATTCAAACACGC-3'

Supplementary Figure.1

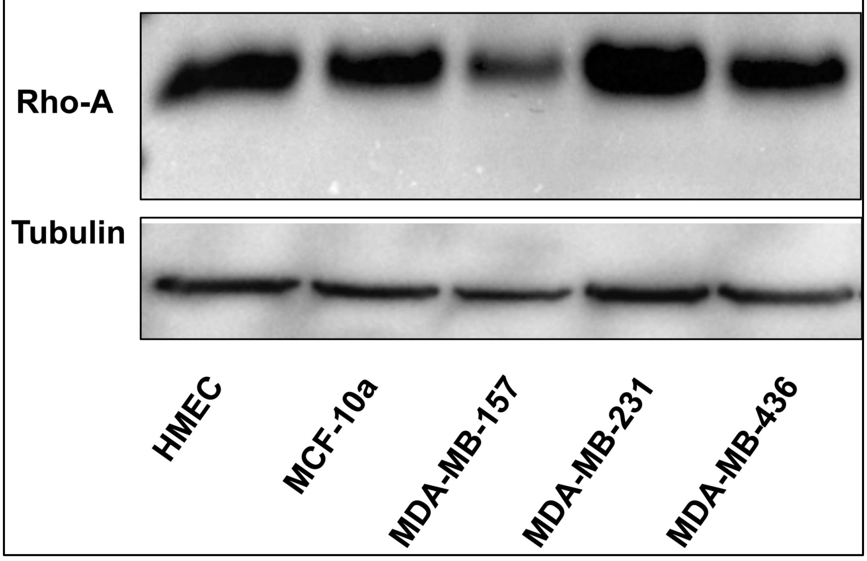

Supplementary Figure.2

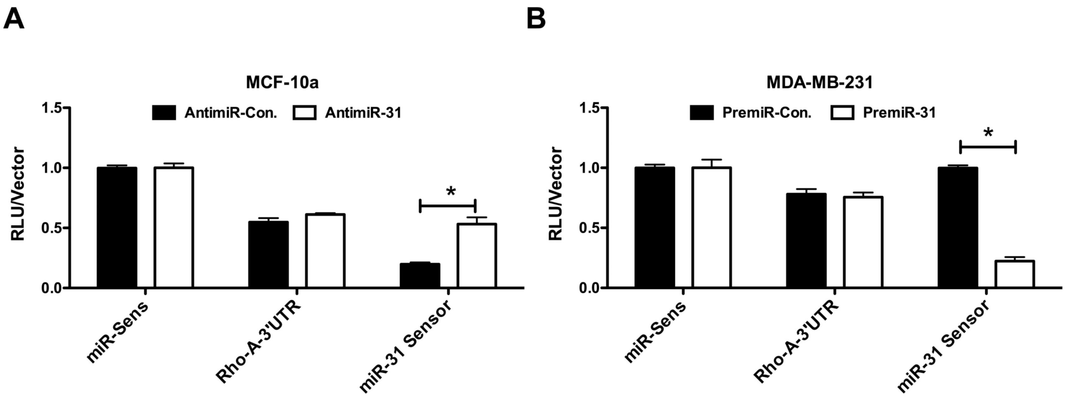

Supplement: Additional file 1: — Table S1. Primers used in the cloning of GNA13-3′-UTR and for site-directed mutagenesis of miR-31 binding sites within the UTR. Oligos used to clone miR-31-Sensor in miR-Sens vector as described in Beillard et al. Figure S1. RhoA protein expression in breast cancer cells: RhoA protein expression showed no correlation to basal miR-31 expression in breast cancer cells (see Figure 3C for miR-31). Immunoblot analysis of RhoA protein levels in a panel of five different breast cell lines is shown. Tubulin was used as loading control. Figure S2. microRNA-31 did not impact RhoA-3′-UTR: (A) Reporter assays performed in MCF-10a cells transfected with antimiR-control or antimiR-31. (B) Reporter assays performed in MDA-MB-231 cells transfected with premiR-control or premiR-31. All reporter assays results are reported relative to miR-Sens-vector treated with antimiR-control or premiR-control respectively. miR-31 sensor is used as a measure of miR-31 activity with and without premiR-31 and antimiR-31 respectively (*, p < 0.05). [file 12943_2015_337_MOESM1_ESM.pdf]
